# Better Data from AI Users: A Field Experiment on the Impacts of Robot Self-Disclosure on the Utterance of Child Users in Home Environment

**DOI:** 10.3390/s23063026

**Published:** 2023-03-10

**Authors:** Byounggwan Lee, Doeun Park, Junhee Yoon, Jinwoo Kim

**Affiliations:** HCI Lab, Business Hall of Yonsei University, Seoul 03722, Republic of Korea; badprince1@gmail.com (B.L.); doeun14@naver.com (D.P.); papong1318@gmail.com (J.Y.)

**Keywords:** human AI interaction, smart speaker, robot utterance, self-disclosure, multi-robot, children

## Abstract

Data are one of the important factors in artificial intelligence (AI). Moreover, in order for AI to understand the user and go beyond the role of a simple machine, the data contained in the user’s self-disclosure is required. In this study, two types of robot self-disclosures (disclosing robot utterance, involving user utterance) are proposed to elicit higher self-disclosure from AI users. Additionally, this study examines the moderating effects of multi-robot conditions. In order to investigate these effects empirically and increase the implications of research, a field experiment with prototypes was conducted in the context of using smart speaker of children. The results indicate that both types of robot self-disclosures were effective in eliciting the self-disclosure of children. The interaction effect between disclosing robot and involving user was found to take a different direction depending on the sub-dimension of the user’s self-disclosure. Multi-robot conditions partially moderate the effects of the two types of robot self-disclosures.

## 1. Introduction

The key general elements of artificial intelligence are data, algorithms, and computing power. As global IT companies build cloud platforms, such as TensorFlow (https://www.tensorflow.org/ (accessed on 1 October 2022)), AWS (https://aws.amazon.com/ (accessed on 1 October 2022)) and Azure (https://azure.microsoft.com/ (accessed on 1 October 2022)), algorithms and computing power grow to such a level that end users can hardly distinguish performance differences. However, data are still fiercely competitive, and depending on the type of data available, the services to which artificial intelligence is applied may differ. Collecting high-quality data is becoming an important factor in the success of artificial intelligence services [1].

However, even in the context of the use of smart speakers, the most popular AI service robot, collecting quality data seems difficult. Smart speakers are artificial intelligence service robots that are operated through a voice user interface (VUI). According to a Nielsen study [2], 24% of US households use smart speakers, and 40% of them own multiple smart speakers. Voice data are considered to be important data in various artificial intelligence fields, such as emotion recognition [3,4,5,6,7]. However, the smart speakers are currently mainly used for simple voice command execution and offer only short and repetitive interactions, such as streaming music and checking the weather [2]. In this situation, the voice data acquired are limited in terms of inferring the emotional or cognitive state of the user.

We believe that if smart speakers could collect better user voice data, artificial intelligence could become more beneficial to our homes, especially for families whose children spend a lot of time at home. According to 2011 census data, more than 4 million children in the United States were alone at home for more than 6 h per week. In Korea, about 30% of elementary school students were alone at home without their parents for more than an hour a day after school [8]. The child may feel anxiety and loneliness due to the fact that they are not provided with the opportunity to interact with family members. Parents also feel anxiety and guilt due to the fact that they do not know the cognitive or emotional state of their children who are home alone [9,10,11]. These feelings are caused by time and space that inevitably disconnect parents and children. To connect parents and children in time and space, it is important for parents to know the current state of the child who is alone in an empty house. We believe that this role could be performed by the smart speakers that are always in the home, but as mentioned above, the amount and quality of data collected so far are not sufficient.

To solve these problems, the quantity and quality of the child’s voice data must be increased. Increasing the quantity and quality of voice data requires that the length of the child’s speech be increased and that the voice contain revealing content, such as thoughts or feelings. In short, this represents an increase in user self-disclosure [12].

Therefore, our research purpose is to elicit user self-disclosure from child users in the context of using a smart speaker, which is representative of an AI service robot. For this purpose, self-disclosure, which has been used as a method to extract the candid story of clients in counseling psychology [13,14,15,16], is adopted in the interaction design of the AI service robot. Two types of robot self-disclosures (disclosing robot utterance, involving user utterance) are proposed to elicit higher self-disclosure from AI users. We examined the effects of these two types of robot self-disclosure on the children’s self-disclosure when using an AI service robot.

Additionally, with recent advances in technology, AI service robots are rapidly entering the same home markets. These trends mean that interactions between humans and AI in the home will not be 1:1 but will involve multiple robots. The number of interaction partners can influence the effects of self-disclosure. Self-disclosure in a 1:N relationship can differ from that in a dyadic relationship. Thus, we also examined the moderating effects of multi-robot conditions.

We conducted a field experiment to investigate the effect of our robot’s utterance strategy. For the empirical experience of children, we developed our own Home Companion-bot, which replicated a smart speaker. We installed our Home Companion-bots in children’s homes to collect realistic measurement data through user experiences in a daily life environment and analyzed the results using these data.

The remainder of this paper is structured as follows: In Section 2, the literature is reviewed and hypotheses are developed. In Section 3, we describe our prototype, Home Companion-bot, and explain how the field experiment was conducted. Section 4 analyzes the results of the experiment. Section 5 discusses the findings and describes the limitations of the study.

## 2. Literature Review and Hypotheses

### 2.1. Self-Disclosure

Self-disclosure has been studied based on a concept from the field of social psychology and further studied in various fields such as communication and counseling psychology [12,15,17,18,19]. Jourard (1958), who initiated a systematic study of self-disclosure, defined self-disclosure as “the process of making self-disclosure to others,” which is an important factor in maintaining the relationship between people in intimate relationships and also a source and indicator of mental health [17].

In the field of clinical and counseling psychology, self-disclosure is defined as “the process by which one person lets his or her inner being, thoughts, and emotions be known to another,” which is important for psychological growth in individual and group psychotherapy [20]. In addition, studies have been conducted on the role of self-disclosure in bringing about discovery and treatment of psychological distress [15,16,21,22,23,24].

In the communications field, self-disclosure is defined as “a process of communication by which one person reveals information about himself or herself to another” [12]. The information can be descriptive or evaluative, and can include thoughts, feelings, aspirations, goals, failures, successes, fears, and dreams as well as one’s likes, dislikes, and preferences [12]. The role of self-disclosure in the development, maintenance, and disappearance of relationships has been studied [25,26,27,28,29,30].

In the field of social psychology, self-disclosure is defined as “any information about oneself” [18,31] in a broad sense. More specifically, self-disclosure is defined as “the verbal communication of personally relevant information, thoughts, and feelings to another” [32] and is often referred to as “willful disclosures that provide insights into personal thoughts and feelings” [33,34], including the actor’s intentions. Studies of the role of self-disclosure in personality factors and individual differences have been conducted [19,35,36,37,38].

Based on these prior studies in sociology and psychology, we have operationally defined self-disclosure as “revealing thoughts and feelings by voice interaction between child users and smart speakers” in this study.

### 2.2. Eliciting Self-Disclosure from Users

#### 2.2.1. Limitations of Prior Studies and Motivations for the Current Study

We reviewed previous studies in the human computer interaction (HCI) or human robot interaction (HRI) field that aimed to elicit users’ self-disclosure. Previous studies examined the interaction factors of the computer or robot that affected users’ self-disclosure, mainly from three perspectives: identity, character traits, and response.

The first aspect is the identity of the interaction partner. The type of interaction partner identity that has an advantage in eliciting self-disclosure is the anthropomorphized entity (virtual humans, avatar interviewers, agents) rather than the human identity [39,40,41,42]. The second aspect was the character traits of the interaction partner. As in interpersonal relationships, character traits—such as prompting effort, likeability, and expressivity—increase the self-disclosure of users [43,44,45]. The third aspect was the response of the interaction partner. The response of an artificial entity positively affects users’ self-disclosure [46,47]. The contents of previous studies are summarized in Table 1 below.

Summing up the review of previous studies, three limitations were identified, which led to our study’s motivation. First, factors such as prompting effort, likability, and expressivity derived from previous studies can be facilitators in eliciting self-disclosure. However, in the context of using smart speakers, interaction with the user is performed through only a short voice. Therefore, there is a need for a more direct way to elicit user self-disclosure. Secondly, the response of an artificial entity such as PPR [46], humanlike nonverbal behavior, and robot-specific nonverbal behavior [47] are passive factors that do not work without user’s utterance. Therefore, it is necessary to design interaction that induces user utterance. Finally, the design factors of most previous studies were manipulated using the Wizard of Oz method without considering whether they would actually work with an AI service robot, so we need a study to investigate self-disclosure from a practical viewpoint.

In order to overcome the limitations of previous studies, we propose a more direct and active interaction method based on counseling psychology, apply this interaction method to the prototype of the AI service robot that actually interacts with child users, and examine the specific effect through a field study in the home environment.

#### 2.2.2. Eliciting Self-Disclosure in Clinical and Counseling Psychology

Counseling psychology is one of the most disciplined fields of study for trying to elicit the inner self of a partner in the context of interpersonal relationships. During counseling, the counselor and the client must establish an intimate relationship so that effective counseling is possible. In order to establish an intimate relationship, the client must trust the counselor, and through this trust, the client should be able to talk comfortably about their problem. For the client to talk about what the they want to say means that the client has been able to perform their own disclosure [22,48]. The self-disclosure of the counselor is an important factor in the self-disclosure of the client. Although self-disclosure of counselors is a controversial issue in terms of achieving the goals of treatment and counseling, self-disclosure of counselors is used as an important technique for gathering a client’s status and information [13,14,15,16].

The self-disclosure methods of counselors can be divided into several types. Hoffman-Graff (1977) examined the effects of therapist self-disclosure on clients by distinguishing them as positive vs. negative [49]. DeForest and Stone (1980) classified self-disclosure according to intimacy level [50]. McCarthy and Betz (1978) classified counselors’ self-disclosure into self-involving statements and self-disclosing statements [51]. Among the different classifications, the method of classifying by self-involving statements and self-disclosing statements is known to be an effective strategy [16].

The therapist’s self-disclosing statement is considered factual information on the part of the therapist about themselves [52] or is referred to as the past history or personal experiences of the counselor [51]. That is, the counselor tells the client about the counselor themselves. On the other hand, the therapist’s self-involving statement is referred to as “the helper’s personal response to statements made by the helpee” [52] or “direct present expressions of the counselor’s feelings about or reactions to the statements or behaviors of the client” [51]. That is, the counselor is talking about their thoughts or feelings about the client.

We applied the above psychological counseling situation to the usage context of the AI service robot. At this time, the role of a therapist—which is to elicit the user’s self-disclosure—can be assigned to an AI service robot, and the role of the client—which is to reveal their inner self—can be assigned to the user. We believe that the AI service robot’s utterance strategy could adopt the use of the self-disclosing statements and the self-involving statement methodology, which have been effective in counseling psychology.

The AI service robot can detect the surrounding situation using sensors and communication networks and can generate speech that provides information to the user based on the detected data. During the exchange, the detected data can be recognized as both the data experienced by the AI service robot and the data experienced by the user within the same space. For the above reasons, we named the self-disclosing statements disclosing robot utterance in the context of the AI service robot usage. The operational definition of the disclosing robot utterance is as follows: the AI service robot utters something to the user about its own experience based on the detected data. Similarly, we named the self-involving statement as involving user utterance, and the operational definition of the involving user utterance is as follows: the AI service robot utters about the user’s thoughts and feelings rather than its own.

#### 2.2.3. Hypothesis of Robot Self-Disclosure Strategies

Social penetration theory [53] argues that interpersonal communication moves from a relatively shallow, non-intimate level to a deeper, more intimate level as a relationship develops. This theory is used to understand the interaction of two individuals in an intimate relationship, explaining that the process occurs through self-disclosure. The intimacy between two people develops through continued sequential exchanges of self-disclosure from superficial to intimate phases, and the self-disclosure at this time occurs reciprocally [54]. In other words, the self-disclosure of the speaker has the effect of inducing the other’s self-disclosure [55].

The reciprocal effect of self-disclosure can be expressed not only in human relationships but also in AI service robots and human interactions. When the self-disclosure of emotions or personality traits are included in the conversational agent’s utterance, the user becomes more aware of the social presence of the artificial agent [56] and feels that it is a conversation with a person [57]. Moreover, the interaction with artificial agents may further facilitate the user’s self-disclosure [58], and the artificial agent’s self-disclosure may play an important role in forming a rapport with the user [59,60,61]. This can be regarded as an effect of anthropomorphism [62,63], which gives human characteristics to non-human agents. Studies in the computers are social actors (CASA) field also argue that users apply social rules of interpersonal relationships to human–computer interaction [64]. Moreover, when computers show characteristics that are similar to human behavior, people are more likely to respond with social behaviors [65,66].

The AI service robot’s detection of surrounding situations using its sensors or networks can be seen as the same as a human’s recognition of surrounding situations using their own senses. The utterances of AI service robots based on these sensing data can be interpreted as the robots’ self-disclosure. At this time, the self-disclosure of AI service robots can be performed by the robots uttering their thoughts and emotions about themselves to the user. During the exchange, the process by which the AI service robot exposes its thoughts and emotions to the user as if it had experienced them itself can be interpreted in the same context as a self-disclosing statement [16,67] in an interpersonal relationship, and this can be defined operationally as disclosing robot utterance.

For the above reasons, it can be said that by expressing information about the home-related situation to the user as if it had happened to the robot, it would generate the disclosing robot utterance, which might increase the self-disclosure of users who are on the receiving end of the interaction, such as the reciprocal effect of self-disclosure in interpersonal relationships. Therefore, we have the following hypothesis:

**H1:**  *The disclosing robot utterance strategy of AI service robot has positive effects on the user’s self-disclosure in a smart speaker context.*

The self-disclosure of the AI service robot could also be performed in such a way that the robot utters its own thoughts and emotions about the user to the user and can be defined as an involving user utterance. As mentioned above, an involving user utterance is a type of self-disclosure that has the same properties as do interactions between people, and the self-disclosure of the user—who receives the disclosure of their interaction partner—can be enhanced by reciprocity. In addition, involving user utterance can be seen as the responsiveness of a partner who understands the situation of their partner in interpersonal communication, and expresses interest and support [68]. Partner responsiveness can enhance the expressivity of communication partners [69]. Therefore, Hypothesis 2 is as follows.

**H2:**  *The involving user utterance strategy of AI service robot has positive effects on the user’s self-disclosure in a smart speaker context.*

According to the interpersonal process model of intimacy, the formation of intimacy consists of self-disclosure and partner-responsiveness [68]. Partner responsiveness is defined as understanding a partner’s experience and position, accepting and respecting the partner’s position and emotions as is, and expressing interest and support for the partner. Mutual intimacy can be formed between the speaker and the listener through the speaker’s expression of their thoughts, emotions, and factual information to the listener (self-disclosure) and the listener’s interaction with the speaker in sympathetic response (partner responsiveness). Self-disclosure is an expression of the feelings and thoughts of the speaker, and partner responsiveness is giving the impression that the listener is understanding, caring, and accepting. The lack of any of these two factors can block the development of intimacy, and it is essential for the development of intimacy that self-disclosure and partner responsiveness are simultaneously achieved.

Applying these two factors to our AI service robot research context, self-disclosure can be regarded as a disclosing robot utterance. This expresses the robot’s own facts, thoughts, and emotions about the surrounding situation. Partner responsiveness is also expressed under involving user utterance conditions. An involving user utterance is defined as the AI service robot talking about the robot’s thoughts about the user, which includes understanding the partner’s situation, accepting the partner’s emotions, and expressing interest. In other words, an involving user utterance is the simultaneous partner responsiveness of a robot that is generated without a user’s previous utterance.

The development of this intimacy implies a shift toward a more intimate relationship in the previously mentioned social penetration theory [53]. A higher intimacy level leads to a higher level of self-disclosure [70]. This means that intimacy can induce a higher level of self-disclosure than a simple reciprocal exchange of self-disclosure with a sense of duty to return something, which was mentioned in social exchange theory [71]. This can be further supported by a study conducted by Rotenberg and Chase (1992) [72]. They found that the reciprocity of self-disclosure in children is covariant with the partner’s intimacy level, which means that the response to a partner’s self-disclosure increases with the level of intimacy. Therefore, if the utterance of the AI service robot satisfies both disclosing robot utterance and involving user utterance conditions, it can be seen as satisfying the conditions for enhancement of intimacy. Improved intimacy will elicit more of the user’s self-disclosure. Therefore, our Hypothesis 3 is as follows:

**H3:**  *Through simultaneous execution of disclosing robot utterances and involving user utterances, the AI service robot’s utterance strategy has an amplifying effect on the user’s disclosure.*

### 2.3. Multi-Robot Systems

#### 2.3.1. Theoretical and Practical Perspectives of Introducing Multi-Robot Systems

The number of interaction partners can influence the effects of self-disclosure. In other words, self-disclosure in a 1:N relationship can differ from that in a dyadic relationship. However, because many previous studies have been carried out under the premise of dyadic interpersonal relationships, such as counseling [16,53,54,55,67,68], there is a lack of studies about eliciting user self-disclosure in a 1:N interaction situation with multiple sources, except for a few studies on group size (e.g., [73]).

The impacts of multi-robot systems can be either positive or negative. From the viewpoint of information processing, an interaction with multiple sources is likely to be perceived more positively than one with a single source in terms of social presence, perceived expertise, attitude, and perceived information quality [74,75,76,77,78]. On the other hand, exchanging information about individuals with multiple entities may increase privacy concerns [79].

Recently, AI service companies have attempted to introduce multiple robots in an individual home by entering the home in a variety of ways. Google Home (https://store.google.com/product/google_home (accessed on 1 October 2022)) and Apple HomePod (https://www.apple.com/homepod/ (accessed on 1 October 2022)) are trying to extend their artificial intelligence services to physical services in the home. At Amazon, Alexa, an artificial intelligence platform, is actively trying to place an Echo series in every room in the house (https://www.amazon.com/Amazon-Echo-And-Alexa-Devices/b?ie=UTF8&node=9818047011 (accessed on 1 October 2022)). Consumer electronics makers such as Samsung and LG are also applying artificial intelligence assistants such as Bixby (https://www.samsung.com/global/galaxy/apps/bixby/ (accessed on 1 October 2022)) and ThinQ (https://www.lg.com/us/lg-thinq (accessed on 1 October 2022)) competitively to all home appliances. In addition, telecommunication companies are competitively launching multiple smart speakers linked to their services at home.

According to the above trends, it is highly probable that multiple AI service robots will be installed in the home in a near future. Users will interact in a 1:N relationship with the multi-robot systems. However, as mentioned earlier, there is little research in sociology or psychology that deals with the effects of self-disclosure in a 1:N relationship. Therefore, in order to theoretically extend self-disclosure research to 1:N interactions and to broaden the practical implications of our robot self-disclosure strategies, it is necessary to examine the effect that multi-robot conditions have on eliciting user self- disclosure.

#### 2.3.2. Hypothesis of Multi-Robot Systems

Privacy concerns are becoming an important issue as AI service robots, such as Amazon Echo and Google Home, come into the home [80,81]. This begins with users’ natural doubts about strangers, such as “Is my smart speaker secure?”, “Is it listening to my conversations?”, and “Where is my voice data stored?”

The level of privacy concern increases from the teens to the late twenties, peaking, and then steadily decreasing until the eighties [82]. It is also known that privacy protection behavior tends to decrease as age increases [83,84,85], while younger users are better at taking protection behavior [83]. Moreover, privacy concerns are increasing in the context of using intelligent voice assistants, such as smart speakers [83]. On the other hand, some studies show that younger users have a lower level of privacy concerns than older people [86]. However, this tendency is a limited case because younger users place more weight on benefits in evaluating the risks and benefits of providing private information [87]. Although previous studies did not subdivide the young user group into children and young adults, privacy concern is an important factor in the context of teenage smart speaker use.

According to Solove (2006) [88], privacy is threatened by three key information-related activities: information collection, processing, and dissemination. Information collection is a process by which a data holder collects and stores data about an individual. Information processing refers to combining, manipulating, or using already-collected data. Information dissemination refers to the transfer or release of the collected information to another person. For each information-related activity, there are various behaviors that may pose a threat to privacy. Increasing the access paths of these activities may increase privacy concerns.

In multi-robot conditions, the user talks to robots about their thoughts and feelings, which means that the information collection path increases without indicating the ultimate use of the information. The increased information collection path further increases privacy concerns, as it is one of the key information-related activities that threaten the privacy of personal information mentioned by Solove (2006) [88].

Increased privacy concerns are known to negatively affect trust [89,90], which may lead to psychological resistance. This decrease in trust and the increase in psychological resistance may negatively affect the reciprocity of the self-disclosure. In particular, this negative effect may be greater when the disclosing robot utterance is executed under multi-robot conditions. The interaction in which several robots express their thoughts or feelings about themselves and induce the self-disclosure of users can be said to be similar to the form in which each client performs self-disclosure in group counseling. Just as clients are hesitant to reveal themselves due to privacy concerns in group counseling [91], the user’s self-disclosure can be reduced if the disclosing robot utterance is performed under multi-robot conditions. Therefore, our Hypothesis 4 is as follows:

**H4:**  *The effect of disclosing robot utterance of AI service robot on user’s self-disclosure is reduced in multi-robot conditions in comparison to that in single-robot conditions.*

According to the reciprocity norms, self-disclosure causes the recipient to feel that they are receiving trust and favor from the partner, and so they offer a similar level of self-disclosure to their partner as a sign of corresponding trust and favor [92,93]. Therefore, people feel uncomfortable when they are exposed to their partner, which means they feel more imbalanced when they think they have revealed more information than their partner. From the viewpoint of social exchange theory [71], the information that one party exposes to another party during the interaction can be seen as cost, and the information to be provided from the other party back to them can be seen as a reward. The imbalance between reward and cost must be corrected, and one way to achieve this is to disclose information of the same perceived value to the partner [94,95]. In particular, this effect may be greater when the involving user utterance is executed under multi-robot conditions. In the involving user utterance, all AI service robots talk about the user, but the user does not feel that reward is increased because the user repeatedly hears the stories they know, as individual robots perform similar roles and functions. As a result, the involving user utterance under multi-robot conditions does not increase the reward but increases the cost. Therefore, increased cost may negatively affect the reciprocity of self-disclosure, and the user will reduce self-disclosure to reduce their own cost. Therefore, our Hypothesis 5 is as follows:

**H5:**  *The effect of involving user utterance of AI service robot on user’s self-disclosure is reduced in multi-robot conditions in comparison to single-robot conditions.*

## 3. Methods

### 3.1. Prototype

To examine the proposed hypotheses, we developed our own Home Companion-bot, a prototype which replicated a smart speaker. The main functions of the Home Companion-bot are as follows.

1. The Home Companion-bot continuously performs ambient sensing for temperature, humidity, illumination, and weather;
2. The Home Companion-bot recognizes the approaching user and greets them with voice;
3. The Home Companion-bot performs self-disclosure to the user based on the detected data. Explanations of stimuli for self-disclosure are explained in detail later in Section 3.5;
4. The Home Companion-bot listens to the user’s response to its self-disclosure and records it. Then, the Home Companion-bot responds to it.

**Figure 1:**
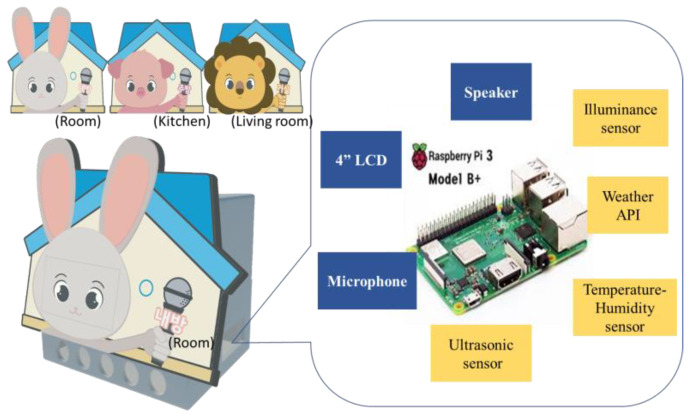
Home Companion-bot.

The hardware was based on Raspberry Pi 3. We used an illuminance sensor (GL5537), a temperature-humidity sensor (DHT22) and the OpenWeatherMap API (https://openweatherermap.org/api (accessed on 1 October 2022)) for ambient sensing. An ultrasonic sensor (HC-SR04P) was used to recognize the approaching user. Microphones (MK1220UB) and speakers (EYEZONE21 EZ-Q1) were used for voice input and output, respectively. A 4-inch HDMI LCD display was used to implement the facial expression of the Home Companion-bot. The software was developed using Python 2.7. The case for the hardware parts was made using a 3D printer and the appearance was designed as a depiction of a familiar animal (rabbit, lion, pig). In multi-robot conditions, three Home Companion-bots were installed in different spaces. A rabbit-bot was installed in the child’s room, a pig-bot was installed in the kitchen, and a lion-bot was installed in the living room. A prototype of the Home Companion-bot is shown in Figure 1.

Home Companion-bots are in a standby state until the participant approaches, with the screen showing the character dozing off. When the participant approaches, the Home Companion-bot goes into an active state, and the conversational turn-taking is shown in the example below.

*Companion-bot’s utterance: Hi, Mary! You look a little stuffy. I think it’s because the sky outside the window is cloudy and dark. Mary, how well do you see outside the window now?*

*Participant’s utterance: I can’t see well out of the window, but my mood is not bad. I played with my friends earlier. So, to be honest, I feel good.*

*Companion-bot’s response: Oh really! Thank you for telling me, Mary.*

Companion-bot’s response consisted of phrases for the natural ending of the conversation. One of the phrases that could normally be used to end a conversation was played randomly, such as “Oh really! Thank you for telling me,” “OK! Tell me again later,” “Oh~ you really did, thank you for telling”. In order to examine only the user’s response corresponding to the self-disclosure of the robot, experiment control was necessary. Therefore, the initiative of dialogue was given to the robot, and the robot’s response to the participant’s speech on the new subject was not implemented.

### 3.2. Pilot Test

Since the experiments were to be conducted in the participants’ homes, it was important to ensure that the Home Companion-bots were working properly, and that the experimental procedure was appropriate. In addition, it was necessary to confirm whether the robot self-disclosure is appropriate and the validity of the measurement item. For this purpose, a pilot test was conducted with two participants.

The two participants were children of double-income households, who spent time at home alone. One participant was an 11-year-old female student who participated in the multi-robot conditions. Another participant was a 15-year-old boy who participated in the single-robot conditions. Three Companion-bots were installed in the participant’s home in multi-robot conditions (child’s room: Rabbit-bot; living room: Lion-bot; kitchen: Pig-bot), and one Home Companion-bot was installed in the participant’s house in single-robot conditions (living room: Rabbit-bot). Each participant experienced four experimental conditions according to combinations of two variables (disclosing robot utterance/involving user utterance), each variable having two levels. The procedures and apparatus of the pilot test were similar to the actual experiments. However, additional steps were taken to correct errors and verify operation of prototypes. It was also checked through a brief follow-up interview that the experimental stimulus, measurement questions, and procedures were appropriate. The results of the pilot test were reflected in the actual experiment.

### 3.3. Experimental Design

To examine the proposed hypotheses, we conducted a 2 (high vs. low disclosing robot) × 2 (high vs. low involving user) × 2 (single- vs. multi-robot interaction) factorial mixed-design experiment. The two independent variables (IVs), disclosing robot and involving user, were used with a within-subjects design, and the moderating variable (MoV), single-/multi-robot, was used with a between-subjects design (see Table 2). In the case of the within-subjects design, the order in which participants experienced each condition group was counterbalanced across participants. In the case of the between-subjects design, participants were randomly and almost equally assigned to each of the two conditions.

### 3.4. Participants

The minimum required sample size was determined using G-power (with a power of 0.80 and alpha of 0.05) [96] and previous studies of the same experimental design [97,98,99]. We required a minimum of 24 participants. The target population in this study was children who spent a lot of time alone at home. Therefore, the participants were children who returned from school to an empty home or children who were left at home with little parental supervision because their parent or parents were away at work. Through elementary school bulletin boards and several online communities of local parents, 31 participants were recruited. Among the participants, there were 19 boys and 12 girls, whose ages ranged from 10 to 14 years (M = 12.26, SD = 1.21). Participants were randomly and almost equally assigned to each of the two between-subject conditions (see Table 2). The experiment was conducted with both the parents’ and children’s consent. After they completed the entire procedure, they received a monetary reward equivalent to approximately USD 45.

### 3.5. Manipulation

Human communication process can be performed simultaneously through two types of messages [100]. One is an explicit message, which is a method of delivering a message directly to a speaker through language. The other is an implicit message, which contains implicit information about the speaker themselves, such as facial expressions or gestures. Therefore, in this study, the robot self-disclosure was comprised of the voice and facial expressions of the Home Companion-bot.

Under the conditions of high disclosing robot utterance, the Home Companion-bot fabricated content about the robot’s state or situation based on the data detected by the robot and delivered the content to the child through the speaker and display output. Examples of speech output were: “Hi! I’m Bunny! Bunny thinks the house is a little cold. I think I may catch a cold. I want to get into the blanket in the room over there. How cold do you think Bunny is?” At the same time, the Home Companion-bot’s face consisted of an animation pointing at itself, which was displayed on the LCD screen. In other words, the Home Companion-bot told a story about the robot itself to the child.

Under the condition of high involving user utterance, the Home Companion-bot made up content about the child’s state or situation based on data gathered by the robot and delivered the content to the child through the speaker output. Examples of speech output were: “Hi, Mary! Even though you are at home, the temperature is low, so you may catch cold. You seem to feel a little cold, you shivered! How cold are you now, Mary?” At the same time, the facial expressions of the Home Companion-bot consisted of an animation that pointed to the user (child), which was displayed on the LCD screen. In other words, the Home Companion-bot told a story about the user (child) to the child.

Low disclosing robot utterance and low involving user utterance conditions were the control conditions in which robot self-disclosure was minimized. The Home Companion-bot output a dummy message, such as a short recorded news clip in an announcer’s voice. The choice of news recorded with the voice of the announcer was intended to minimize the robot self-disclosure with a voice that was different from the Home Companion-bot’s voice (from pilot test results). At the same time, the Home Companion-bot’s facial expression consisted of an expressionless appearance, and this was shown on the LCD screen.

**Figure 2:**
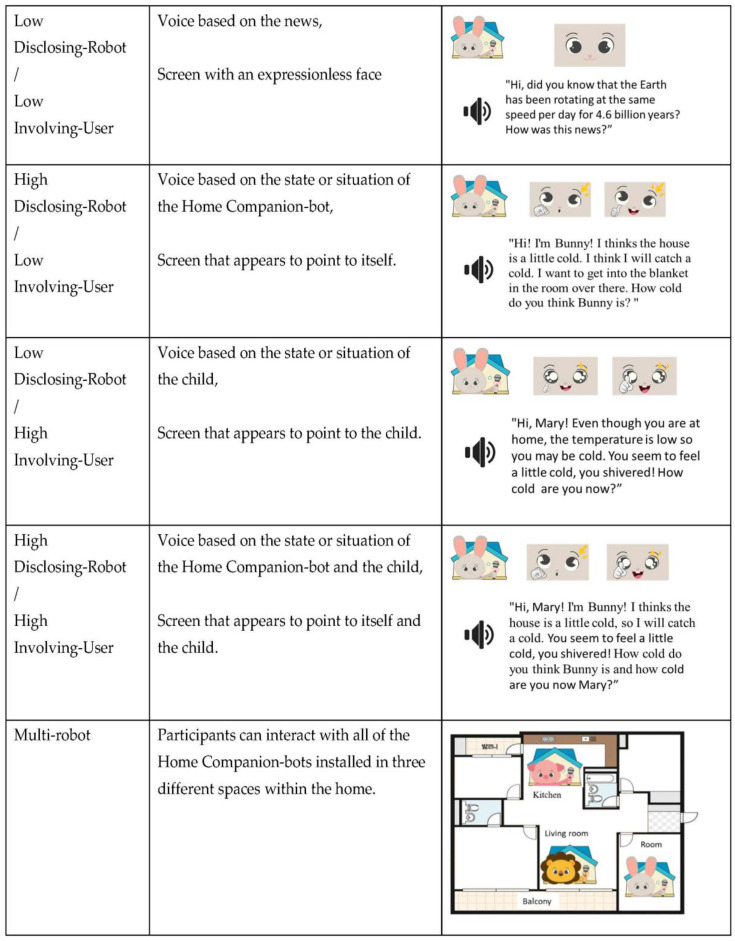

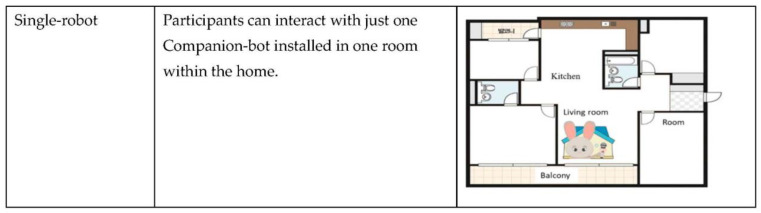
Manipulation of disclosing robot, involving user, and multi-robot conditions.

In multi-robot conditions, each of the robots used a different text-to-speech (TTS) engine according to their appearance, so different voices were output. NAVER’s TTS engine “Jinho,” “Mijin” (https://clova.ai/voice/ (accessed on on 1 October 2022)), and Google’s TTS engine (https://cloud.google.com/text-to-speech/docs/basics (accessed on on 1 October 2022)) were used. The robots’ utterances also consisted of content that fit the unique spaces of the living room, kitchen, and the child’s room to enhance the implementation of the multi-robot conditions (from pilot test results). Examples of speech output were as follows: child’s room (Rabbit-bot): “Hi! I’m Bunny! I think it’s a little cold now. Sadly, I think I’ve got a bit of a cold. I think it’s better to turn on the heating. How cold do you think Bunny is?”; kitchen (Pig-bot): “Hi! I’m Piggy! From a while ago, it seems a little cold. I want to boil warm barley tea in a kettle. I feel a little chilly. How cold do you think Piggy is?”; living room (Lion-bot): “Hi! I’m Lion! I think the living room is a bit cold. I think I have a cold. I want to get into the blanket in that room. How cold do you think Lion is?” In single-robot conditions, Rabbit-bot was used with NAVER’s TTS engine “Mijin”. A summary of the experimental conditions is presented in Figure 2.

Since the subject of the contents must be different for each type of robot self-disclosure, the text length of the robot utterances is not exactly the same. For example, in greeting messages, the difference in the text length was inevitable; “Hi! I’m Bunny!” (in the conditions of high disclosing robot and low involving user), “Hi, Marry!” (in the conditions of low disclosing robot and high involving user), “Hi! Marry! I’m Bunny.” (in the conditions of high disclosing robot and high involving user). However, in other contents, it is designed to be composed of similar characters.

### 3.6. Procedure

Prior to the experiment, it was necessary to prevent children from feeling uncomfortable about the sudden self-exposures of robots when they experienced it for the first time [101]. To solve this problem, a Home Companion-bot introduction video was sent to the participants one or two days in advance. Additionally, it was necessary to collect realistic measurement data through user experiences in a daily life environment. For this reason, the experiments were performed at the participants’ homes individually.

The participants and their parents were provided with information about the experimental procedure in writing, and the experiment began with the consent of both the parents and their children. To minimize the novelty effect that had occurred in the pilot test, participants watched a video of the four conditions they would experience before beginning the experiment and used the Home Companion-bot several times.

**Figure 3:**
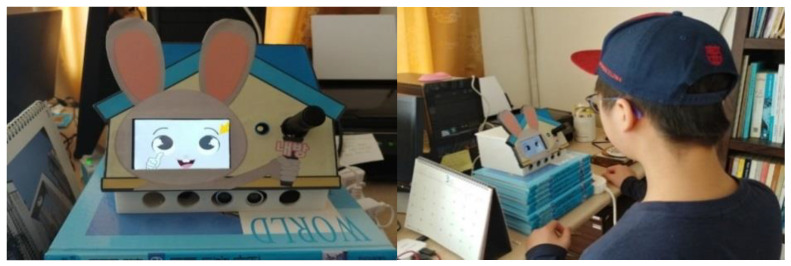
Participants and Home Companion-bot.

While the participants experienced each condition, the experimenter was left out of the participant’s home so that participants could experience the conditions naturally in their daily lives. Participants approached the Home Companion-bot whenever they wanted and experienced experimental stimuli and responded with a voice. This process was carried out about 10 times for each condition, and the time required to experience one condition was approximately 0.5 to 1 h. Participants answered a questionnaire after experiencing each condition. Figure 3 shows the participants and the Home Companion-bot.

### 3.7. Measurements

User self-disclosure is a construct consisting of multiple dimensions. Wheeless and Grotz (1976) and Wheeless (1978) conceptualize these as five dimensions: amount, depth, honesty, intent, and valence [102,103]. Amount represents the frequency and duration of an individual’s disclosures. Depth reflects the degree of intimacy in the communication. Honesty refers to the accuracy with which one communicates information about oneself. Intent reflects an individual’s control and awareness over their self-disclosures. Valence is the positive nature of the information being disclosed in the communication. This conceptualization has been used in other studies on self-disclosure models in the online community and social network services [104,105,106]. Collins and Miller (1994) also discussed self-disclosure as two dimensions of depth and breadth [25]. Depth is referred to as the quality of the information to be exposed and measured by an independent judge. On the other hand, breadth is referred to as the amount of varying information and is measured in words or time. Expanding on this, Moon (2000) added a number of intimate self-disclosures to the above two dimensions, supplementing the quantitative aspect of self-disclosure that was lacking in breadth alone [101]. The study by Hollenbaugh and Ferris (2015) covered three dimensions of self-disclosure: amount, depth, and breadth [107].

Based on the above studies, this study operationalized users’ self-disclosure as a construct consisting of three sub-dimensions in a smart speaker context: speech length, amount, and depth. Speech length was defined as the length of data that the user uttered by voice to the smart speaker. Perceived amount was defined as the degree to which the user perceives how much the user expressed his or her thoughts or emotions to the smart speaker. Perceived depth was defined as the degree to which the user perceives how deeply the user expressed his or her thoughts or emotions to the smart speaker.

Measurement was performed in two ways. First, we measured the speech length of the child responding to the self-disclosure of the Home Companion-bot. It was measured by converting the voice response of the child, recorded by the Home Companion-bot, to text and then counting the number of characters in the text. Amount and depth were measured using questionnaire items. The questionnaire items used to measure users’ self-disclosure were from prior validated studies [108,109]. These items have been revised to the minimum to fit the context of smart speaker usage and translated into Korean. The questionnaire items for the manipulation check were based on the operational definition of the IVs in this study. To check the validity and reliability of the measurements, HCI experts were asked to review the measurement items and a pre-test was conducted with 15 participants. Some of the items were modified or removed to reflect the results of the pre-test. Finally, 14 questionnaires and 1 word count method were used for the measurement. The responses of the questionnaire items were measured on a 7-point Likert scale. The measurement items are shown in Appendix A.

## 4. Results

Considering the characteristics of field experiments conducted in children’s homes, the outlier criteria were: (1) participants are unable to concentrate on the experiment due to an unexpected external situation (e.g., a visit or invitation from a friend) and (2) participants are not interested in participating in an experiment (e.g., participation due to parental coercion). Of the 31 participants, 3 (3 male) in single-robot conditions and 3 (2 male, 1 female) in multi-robot conditions were judged to be outliers, and the data collected from them were excluded from the analysis. The data of the remaining 25 participants were statistically analyzed. Among the participants, there were 14 boys and 11 girls, whose ages ranged from 11 to 14 years (M = 12.32, SD = 1.12). In total, 12 were in single-robot conditions, and 13 were in multi-robot conditions.

### 4.1. Manipulation Check

To verify that the IVs of this study were implemented with appropriate stimuli in the experiment, we performed a manipulation check. The questionnaires for disclosing robot and involving user conditions were measured using the 7-point Likert scale.

For each experimental condition group, averages of the responses were analyzed using paired-sample t-tests. Significantly higher disclosing robot values were reported from the high disclosing robot condition groups ((*M* = 5.95, *SD* = 1.02), (*M* = 5.73, *SD* = 1.31)), compared to the low disclosing robot groups ((*M* = 1.64, *SD* = 0.96), (*M* = 3.31, *SD* = 1.84)). These results were statistically significant (*t*(24) = 14.87, 11.73, 7.63, 5.44|all *p*-values < 0.001). In terms of involving user, the high involving user condition groups ((*M* = 5.66, *SD* = 1.21), (*M* = 5.51, *SD* = 1.26)) reported significantly higher involving user values than the low condition groups ((*M* = 1.55, *SD* = 1.14), (*M* = 2.85, *SD* = 1.44)). These results were also statistically significant (*t*(24) = 13.22, 10.83, 7.18, 7.76|all *p*-values < 0.001).

### 4.2. Measurement Validation

Prior to conducting the hypothesis test, we performed measurement validation. The Smart PLS (v. 3.2.7) software was used. In the convergent validity test results, the factor-loadings and average variance extracted (AVE) were within acceptable ranges (factor-loading > 0.70, AVE > 0.50) [110]. In the reliability test, Cronbach’s alpha and composite reliability values exceeded the recommended threshold of 0.7 [111,112].

Also, the square roots of the AVE for each construct in this study were higher than the inter-construct correlations. Therefore, this study satisfied the condition for discriminant validity [112,113,114].

### 4.3. Hypothesis Testing

**Figure 4:**
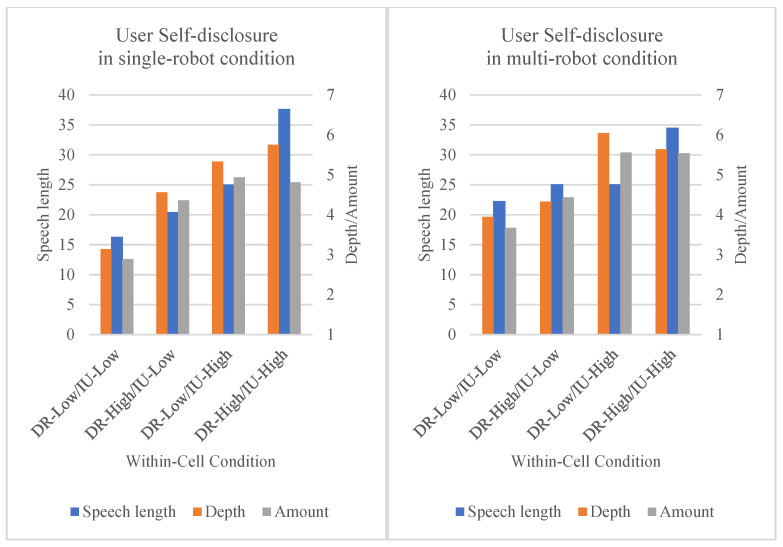
DV measurement results (user self-disclosure). DR: disclosing robot; IU: involving user.

As mentioned above, this study operationalized user self-disclosure as a construct consisting of three sub-dimensions: speech length, amount, and depth. To measure speech length, our prototype Home Companion-bot automatically recorded the voice the child responded with. The recorded voice was then converted to text and the data was collected by counting the number of characters. To measure amount (amount of user Self-disclosure) and depth (depth of user self-disclosure), data were collected by having participants respond to the questionnaire items. Table 3 and Figure 4 show the descriptive statistics (mean value, standard deviation) of the results of each experimental condition.

To analyze the effect of two IVs, disclosing robot and involving user, as within-subject factors on user self-disclosure (speech length, depth, amount), and to analyze the moderating effect of multi-robot as a between-subjects factor, we ran a 2 × 2 × 2 mixed ANOVA. For this, SPSS 24 IBM was used. The effect sizes were calculated using the generalized eta-squared statistic [115].

#### 4.3.1. Speech Length

The results on Speech Length are displayed in Table 4.

**Figure 5:**
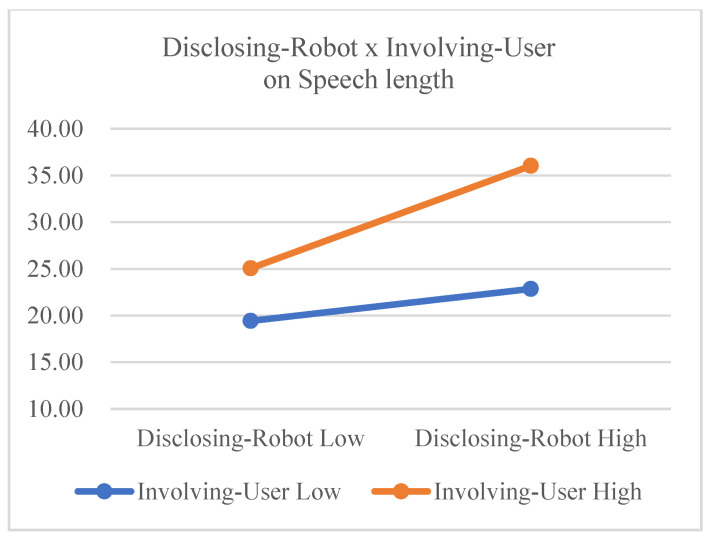
Interaction effects on speech length (between IVs).

The main effect of disclosing robot on speech length was significant (*F*(1,23) = 28.894, *p* < 0.001, η_G_^2^ = 0.256), and the main effect of involving user was also significant (*F*(1,23) = 35.756, *p* < 0.001, η_G_^2^ = 0.374). These results support Hypotheses 1 and 2. The interaction effect between disclosing robot and involving user was significant (*F*(1,23) = 8.735, *p* = 0.007, η_G_^2^ = 0.086). Figure 5 demonstrates that the relationship between disclosing robot and speech length is always positive, but it is far more so for the high involving user condition (orange line) than the low involving user condition (blue line). This result supports Hypothesis 3.

**Figure 6:**
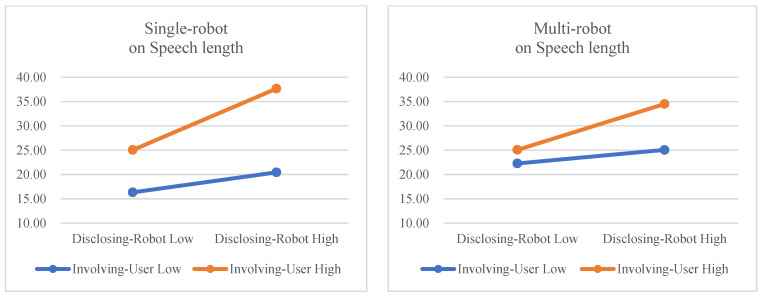
Moderation effects of multi-robot on speech length.

We also checked whether multi-robot showed moderating effects in the relation between IVs (disclosing robot and involving user) and speech length (see Figure 6). The interaction effect between disclosing robot and multi-robot was not significant (*F*(1,23) = 0.699, *p* = 0.412), but the interaction effect between involving user and multi-robot was significant (*F*(1,23) =4.604, *p* = 0.043, η_G_^2^ = 0.071). These interaction effects reveal that the effect of disclosing robot on speech length was not moderated by the multi-robot condition, but the effect of involving user on speech length was reduced in multi-robot conditions. When involving user is low, the single-robot condition has more of an effect on speech length than does the multi-robot condition. This is shown in Figure 6; the gap between the blue line and the orange line is larger in the left picture than in the right picture. These results do not support Hypothesis 4 but do support Hypothesis 5. Finally, the three-way interaction of disclosing robot, involving user, and multi-robot was not significant (*F*(1,23) = 0.131, *p* = 0.721).

#### 4.3.2. Depth

The results on depth are displayed in Table 5.

**Figure 7:**
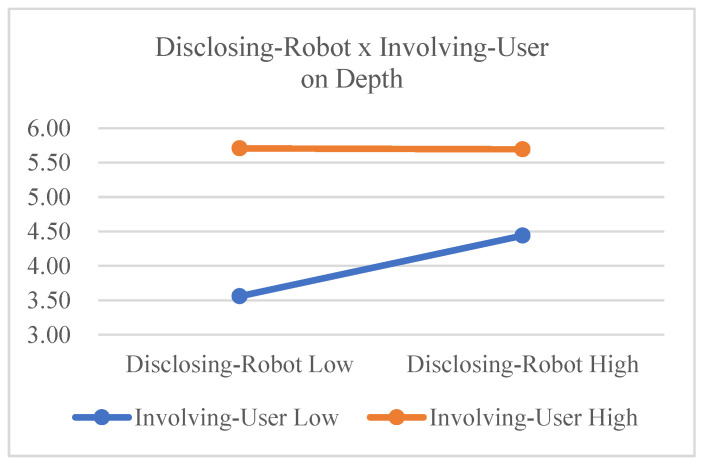
Interaction effects on depth (between IVs).

The main effect of disclosing robot on depth was significant (*F*(1,23) = 4.649, *p* = 0.042, η_G_^2^ = 0.023), and the main effect of involving user was also significant [*F*(1,23) = 31.395, *p* < 0.001, η_G_^2^ = 0.249]. These results support Hypotheses 1 and 2. The interaction effect between disclosing robot and involving user was marginally significant (*F*(1,23) = 3.179, *p* = 0.088, η_G_^2^ = 0.023). From this result, we can infer that the positive effect of disclosing robot on depth (depth of user self-disclosure) is smaller in involving user high conditions than in involving user low conditions (see Figure 7). This result marginally supports Hypothesis 3.

**Figure 8:**
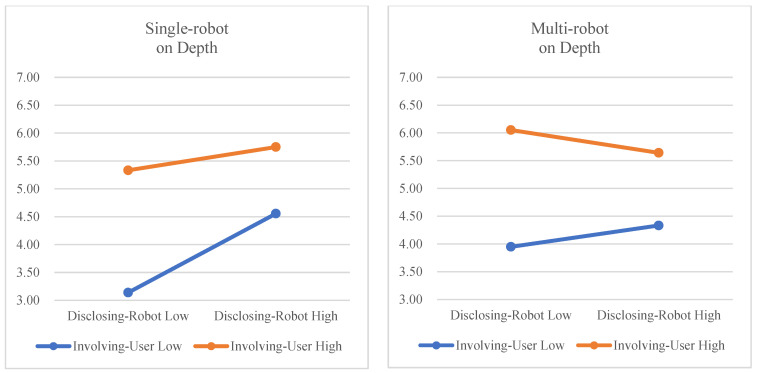
Moderation effects of multi-robot on depth.

We also checked whether multi-robot showed moderation effects in the relation between IVs (disclosing robot and involving user) and depth (see Figure 8). The interaction effect between disclosing robot and multi-robot was statistically significant (*F*(1,23) = 4.916, *p* = 0.037, η_G_^2^ = 0.024), but the interaction effect between involving user and multi-robot was not significant (*F*(1,23) =0.000, *p* = 0.986). Interestingly, this result shows a different trend from the previous speech length analysis. In the speech length analysis, the multi-robot condition reduced the effect of involving user. However, in the depth analysis, the disclosing robot effect was reduced by the multi-robot condition. The effect of disclosing robot on depth was increased by the single-robot condition, but the effect of disclosing robot on depth was reduced by the multi-robot condition. The effect of involving user on depth was not moderated by the multi-robot condition. These results do support Hypothesis 4, but do not support Hypothesis 5. The three-way interaction of disclosing robot, involving user, and multi-robot was not significant (*F*(1,23) = 0.066, *p* = 0.840).

#### 4.3.3. Amount

The results on amount are displayed in Table 6.

**Figure 9:**
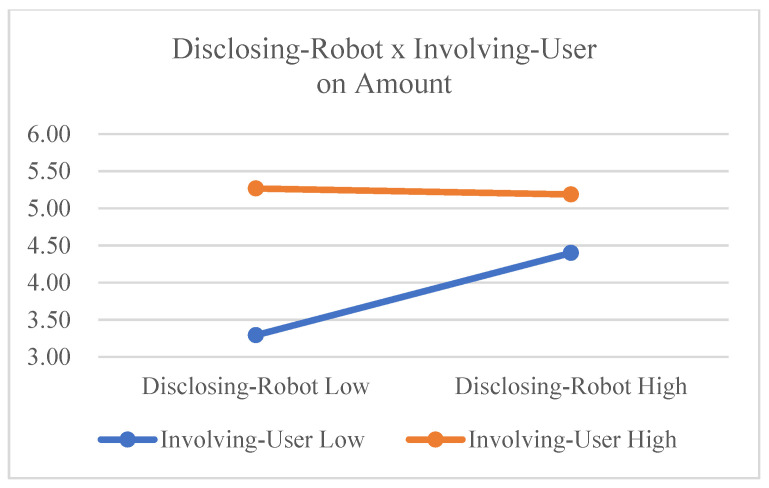
Interaction effects on amount.

The main effect of disclosing robot on amount was significant (F(1,23) = 4.359, *p* = 0.048, η_G_^2^ = 0.037), and the main effect of involving user was also significant (F(1,23) = 24.254, *p* < 0.001, η_G_^2^ = 0.213). These results support Hypotheses 1 and 2. The interaction effect between disclosing robot and involving user was significant (F(1,23) = 7.982, *p* = 0.010, η_G_^2^ = 0.049). However, in Figure 9, the slope of the orange line demonstrates that the positive effect of disclosing robot on amount (amount of user self-disclosure) is smaller in involving user high conditions than in involving user low conditions. These results suggest that the interaction effect is significant but occurs in a different direction, so Hypothesis 3 is partially supported.

**Figure 10:**
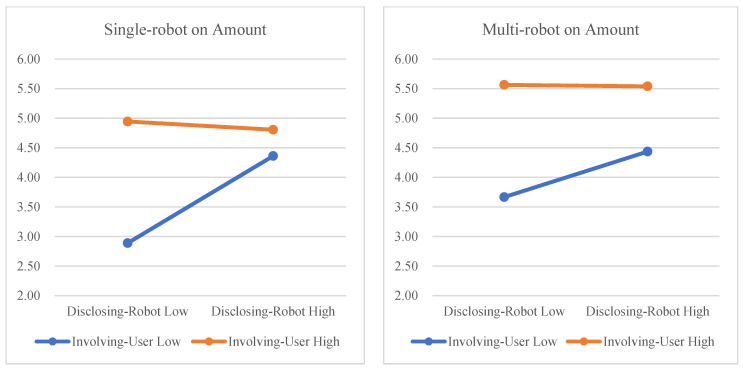
Moderation effects of multi-robot on amount.

We also checked whether multi-robot showed moderation effects in the relation between the IVs (disclosing robot, involving user) and amount (see Figure 10). The interaction effect between disclosing robot and multi-robot was not statistically significant (F(1,23) = 0.351, *p* = 0.559), and the interaction effect between involving user and multi-robot was not significant (F(1,23) = 0.200, *p* = 0.659). These results do not support Hypotheses 4 or 5.

#### 4.3.4. Summary of Hypothesis Testing

Hypotheses 1 and 2 were fully supported because of the significant ANOVA results in all sub-dimensions of the dependent variable (DV). Hypothesis 3 was partially supported, which is consistent with our expectation for speech length, but the direction of the interaction effect was different from its direction in other sub-dimensions. Hypothesis 4 was partially supported because the sub-dimension of DV showed significant results for depth. Hypothesis 5 was partially supported because the speech length of DV’s sub-dimension was significant. Table 7 summarizes the results.

## 5. Discussion

This study proposed “Disclosing-Robot” and “Involving-User” as a robot’s utterance strategy for an AI service robot to elicit self-disclosure from users in the context of a smart speaker for children. To examine the effects of these strategies, we developed our own Home Companion-bot prototypes and conducted a field experimental study in which children participated at home.

As a result of this study, we confirmed that both types of robot utterance strategies were effective in eliciting the self-disclosure of children. Interestingly, the interaction effect between disclosing robot and involving user was found to take a different direction depending on the sub-dimension of the user’s self-disclosure. In terms of the speech length of the child’s utterance, the interaction effect was observed to have the amplified direction, which was consistent with our hypothesis. However, in terms of depth and amount, the interaction effects of the two utterance strategies conflicted with each other, which differed from our hypothesis.

The reason why the interaction effect occurred in the different direction could be inferred from the exploitative properties of TSD (therapist self-disclosing) and TSI (therapist self-involving), such as when the roles of the counselor and the client are reversed in counseling psychology [116,117]. If the counselor’s self-disclosure appears to dominate the dialog situation, or if the counselor appears to be uninterested in listening to the client’s comments, the counselor’s comments can be viewed as insensitive [118]. Additionally, if the counselor makes a very detailed and lengthy disclosure, the client may consider the counselor to be in a subordinate position, such as “chatty,” which reduces the disclosure of the client and causes the client to react negatively to the counselor’s deficiencies [119].

The case in which AI service robots use both utterance strategies (disclosing robot, involving user) may be similar to the above case, in which the self-disclosure of a counselor has exploitive attributes. In this case, the AI service robot exposes both the story of the robot itself and the story of the user at an equal level, hence seeming “chatty”, similarly to a counselor who makes more and more disclosure. The user recognizes the AI service robot not as an object that listens to the user but as a robot that speaks its own thoughts. This may reduce the exposure of the user’s thoughts and feelings. That is, the depth and amount of user self-disclosure are reduced. As a result, the simultaneous occurrence of two robot utterance strategies reduced the depth and amount of user self-disclosure. During this process, the level of intimacy was not reduced, so the speech length has an interaction effect in the amplifying direction that differed from depth and amount.

We also found that the multi-robot condition moderated the effects of the two types of robot utterance strategies. Moderation effects partially affected the sub-dimensions of user’s self-disclosure, and the effect on the sub-dimensions was different according to the type of robot self-disclosure. Specifically, the effect of disclosing robot on depth was reduced in multi-robot conditions compared to single-robot conditions. The effect of Involving-User on Speech length was reduced in multi-robot conditions.

As mentioned in the Section 2.3.2 hypothesis, in multi-robot conditions, the privacy concern increases, which increases user cost in the social exchange process and reduces trust. We had expected that the user self-disclosure would be reduced by the effect of increased cost and reduced trust. However, this effect was different for the two types of strategies.

When disclosing robot was executed under multi-robot conditions, each AI service robot told the user the robot’s own story. The user received the information about the different robots from each robot, and the reward in the social exchange process could be increased. The increased reward offset the increase in cost due to privacy concerns, which may explain why the moderating effect of multi-robot in terms of speech length was not significant. In terms of depth, as we predicted, the decrease in trust seems to have led to doubts about whether the robot revealed its own story, thus reducing the depth of user self-disclosure.

On the other hand, when involving user was executed in multi-robot conditions, all AI service robots talked about the user. At this time, even though the user interacted with several robots, the user repeatedly heard the stories they knew, and the reward in the social exchange process felt by the user did not change. In the unchanged state of reward, the speech length may have been reduced to reduce the cost raised by privacy concerns, as we expected. However, the involving user utterance could also be heard as an estimate of what the user knows. Because of this, even though the trust decreased due to the privacy concerns that occurred in multi-robot conditions, the influence on depth of user self-disclosure was not significant because the user knows the truth anyway.

For both the disclosing robot and the involving user utterances, the moderating effect of multi-robot was not significant to the amount of user self-disclosure, which can be explained as follows. The Home Companion-bot, which was used in this study, was designed to tell the user about one topic created by one of the sensors when performing robot self-disclosure. Additionally, it was designed to have one expression of thoughts and feelings. In other words, the amount included in the self-disclosure interaction process was minimal. Due to this minimal amount, a floor effect may have occurred when the multi-robot condition tried to reduce amount, but it could not be reduced anymore.

### 5.1. Research Implications

This research has various implications both theoretically and practically. The theoretical implications are as follows. First, it is an extension of the robot self-disclosure concept. This study classified robot self-disclosure into two types according to the context of an AI service robot for children. We newly applied and expanded the concept from counseling psychology. Secondly, it has been shown that there is an interaction effect between the two types of robot self-disclosure. Although disclosing robot and involving user have interactive effects that amplify each other’s effects, they have different effects on the sub-dimension of user’s self-disclosure. This can be said to have overcome existing limitations, which can only be confirmed by the main effects of “self-disclosing statement” and “self-involving statement” due to the restricted environment in counseling psychology. Lastly, we have expanded the effect of self-disclosure to a 1:N relationship. Previous studies on self-disclosure have focused mainly on dyadic relationships in counseling. However, this study examined the effects of self-disclosure in the 1:N human–AI relationship. In particular, the important implication is that the multi-robot condition moderates the influence of the robot’s utterance strategy in eliciting the user’s self-disclosure. These results may contribute to expanding the theory of self-disclosure in sociology or psychology to the 1:N interaction perspective.

The practical implications of this study are as follows. First, this study suggests that direct and active utterance strategies of AI service robots increase the user’s self-disclosure. We applied the sensed environment data to the conversation material of the artificial intelligence service robot. This is different from existing computers, which provide information passively at the user’s request. The second is the expansion of the role of AI service robot. In this study, we proposed a voice interaction method that can understand the user rather than only execute commands. AI service robots that better understand users in the home can also act as mediators to convey the status of family members beyond music streaming tools. The last practical implication is the suggestion of how to interact with a multi-robot system. Of course, many service robots with AI technology coming into the house can ease the troubles in our lives and make them more convenient. However, it is considered inappropriate for all robots to try and interact with the user in terms of eliciting self-disclosure from the user (child). In order to obtain rich information from the user, it is necessary to have a strategy using a single robot that initiates interactions with the user.

### 5.2. Limitations

This study has several limitations as well. First, the experimental environment was not fully controlled. Because it was a field experiment conducted at the children’s home, variables such as the presence of people (parents, siblings) in the same room or using other media (TV, mobile phone) could not be controlled. However, the authors tried to provide as realistic environments as possible during the experiment. Secondly, the participants were aged from 10 to 14 years old and from Korea only, so cultural and age factors may have impacted the study results. Although this study was conducted for participants with limited cultures and ages, the primary effects were first confirmed in the target populations. Thirdly, we have not observed the effect of applying robot self-disclosure, which is the result of this research, to actual AI services over a long period of time.

### 5.3. Future work

The direction of future work is to overcome the limitations of this study and to incorporate the interaction design of this study into consumer products. Long-term experiments using multifunctional commercial products will minimize exogenous variables and demographic differences such as gender, age, and culture. In addition, we will explore how accumulated user self-disclosure can contribute to improving the performance of artificial intelligence or creating services for users.

## Figures and Tables

**Table 1:** Prior studies on eliciting self-disclosure.

	Authors	Contents	Results
Identity of the interaction partner [39,40,41,42]	Powers et al. (2007) [39]	Computer agent/Humanoid robot	agent is more effective
	Lucas, et al. (2014) [40]	A virtual human interviewer as computer or human	Virtual humans increase willingness to disclose
	Pickard, Roster, and Chen (2016) [41]	Preferences for partner identity change with information sensitivity	Participants preferred avatar interviewers for more sensitive topics and preferred human interviewers for less sensitive topics
	Kumazaki et al. (2018) [42]	The effect of the android and simplistic humanoid robots on the self- disclosure	Simple robots are more effective on the self-disclosure of ASD adolescents
Character traits of the interaction partner [43,44,45]	Mumm and Mutlu (2011) [43]	Study on how robot likeability affects the psychological distance	The robot’s likeability marginally affected human self-disclosure
	Bethel, Stevenson, and Scassellati (2011) [44]	On the possibility that robots can collect sensitive information of children	Robots’ prompting efforts should be of a similar level to that of adults
	Martelaro, Nenji, Ju, and Hinds. (2016) [45]	Effects of robot vulnerability and expressivity on user trust, companionship and disclosure	While vulnerability increased trust and companionship, expressivity increased the disclosure.
The response of the interaction partner [46,47]	Hoffman et al. (2014) [46]	PPR (perceived partner responsiveness) of a robot	The higher the PPR of a robot, the more self-disclosure people had
	Rosenthal-von der Pütten et al. (2018) [47]	Classified non-verbal behaviors of an artificial entity into HNB (human-like non-verbal Behavior) and RNB (robot-specific non-verbal behavior)	All types of non-verbal behavior increase the breadth of self-disclosure

**Table 2:** Experimental design and number of participants in between-subject groups.

Within-Subjects					
**IV1 (2-level)**		Low Disclosing Robot		High Disclosing Robot	
**IV2 (2-level)**		Low Involving User	High Involving User	Low Involving User	High Involving User
**Between-subjects** **MoV**	Single robot N = 15	S(0,0)	S(0,1)	S(1,0)	S(1,1)
	Multi-robot N = 16	M(0,0)	M(0,1)	M(1,0)	M(1,1)

**Table 3:** Descriptive statistics for speech length, amount, and depth of user self-disclosure.

IVs, MoV Condition			DV (User Self-Disclosure)		
Disclosing Robot	Involving User	Multi- or Single-Robot	Speech Length	Amount	Depth
Low	Low	Multi	22.28 (12.22)	3.67 (1.66)	3.95 (1.74)
		Single	16.33 (7.34)	2.89 (1.58)	3.14 (1.93)
High	Low	Multi	25.07 (9.06)	4.44 (1.48)	4.33 (1.71)
		Single	20.45 (6.04)	4.36 (1.46)	4.56 (1.68)
Low	High	Multi	25.07 (13.43)	5.56 (0.85)	6.05 (0.78)
		Single	25.05 (12.81)	4.94 (1.45)	5.33 (1.44)
High	High	Multi	34.51 (12.57)	5.54 (1.05)	5.64 (1.58)
		Single	37.67 (13.77)	4.81 (1.30)	5.75 (1.15)

Note: Standard deviations are shown in parentheses. IV: independent variable; MoV: moderating variable; DV: dependent variable.

**Table 4:** ANOVA results for speech length.

DV	IVs, MoV	*F* Statistic	Significance Level	Generalized Eta-Squared	Hypotheses Supported (for Speech Length)
Speech length	DR	*F*(1,23) = 28.894	*p <* 0.001 ***	η_G_^2^ = 0.256	H1: Supported
	IU	*F*(1,23) = 35.756	*p <* 0.001 ***	η_G_^2^ = 0.374	H2: Supported
	DR × IU	*F*(1,23) = 8.735	*p* = 0.007 **	η_G_^2^ = 0.086	H3: Supported
	DR × Multi-robot	*F*(1,23) = 0.699	*P* = 0.412	η_G_^2^ = 0.008	H4: Not supported
	IU × Multi-robot	*F*(1,23) = 4.604	*p* = 0.043 *	η_G_^2^ = 0.071	H5: Supported

Note: * *p* < 0.05, ** *p* < 0.01, *** *p* < 0.001. IV: independent variable; MoV: moderating variable; DV: dependent variable; DR: disclosing robot; IU: involving user.

**Table 5:** ANOVA results for depth.

DV	IVs, MoV	*F* Statistic	Significance Level	Generalized Eta-Squared	Hypotheses Supported (for Depth)
Depth	DR	*F*(1,23) = 4.649	*p* = 0.042 *	η_G_^2^ = 0.023	H1: Supported
	IU	*F*(1,23) = 31.395	*p <* 0.001 ***	η_G_^2^ = 0.249	H2: Supported
	DR × IU	*F*(1,23) = 3.179	*p* = 0.088	η_G_^2^ = 0.023	H3: Marginally Supported
	DR × Multi-robot	*F*(1,23) = 4.916	*p* = 0.037 *	η_G_^2^ = 0.024	H4: Supported
	IU × Multi-robot	*F*(1,23) = 0.000	*p* = 0.986	η_G_^2^ = 0.000	H5: Not Supported

Note: * *p* < 0.05, *** *p* < 0.001. IV: independent variable; MoV: moderating variable; DV: dependent variable; DR: disclosing robot; IU: involving user.

**Table 6:** ANOVA results on amount.

DV	IVs, MoV	*F* Statistic	Significance Level	Generalized Eta-Squared	Hypotheses Supported (for Amount)
Amount	DR	*F*(1,23) = 4.359	*p* = 0.048 *	η_G_^2^ = 0.037	H1: Supported
	IU	*F*(1,23) = 24.254	*p <* 0.001 ***	η_G_^2^ = 0.213	H2: Supported
	DR × IU	*F*(1,23) = 7.982	*p* = 0.010 *	η_G_^2^ = 0.049	H3: Partially supported
	DR × Multi-robot	*F*(1,23) = 0.351	*p* = 0.559	η_G_^2^ = 0.003	H4: Not supported
	IU × Multi-robot	*F*(1,23) = 0.200	*p* = 0.659	η_G_^2^ = 0.002	H5: Not Supported

Note: * *p* < 0.05, *** *p* < 0.001. IV: independent variable; MoV: moderating variable; DV: dependent variable; DR: disclosing robot; IU: involving user.

**Table 7:** Summary of results.

Hypotheses	IVs, MoV	DV Sub-Dimension	*p*-Value	Remark	Hypotheses Supported
H1	Disclosing robot	Speech length	*p <* 0.001 ***	O	Supported
		Depth	*p* = 0.042 *	O	
		Amount	*p* = 0.048 *	O	
H2	Involving user	Speech length	*p <* 0.001 ***	O	Supported
		Depth	*p <* 0.001 ***	O	
		Amount	*p <* 0.001 ***	O	
H3	Disclosing robot × Involving user	Speech length	*p* = 0.007 **	O	Partially Supported
		Depth	*p* = 0.088	Different direction	
		Amount	*p* = 0.010 *	Different direction	
H4	Disclosing robot × Multi-robot	Speech length	*p* = 0.412		Partially Supported
		Depth	*p* = 0.037 *	O	
		Amount	*p* = 0.559		
H5	Involving user × Multi-robot	Speech length	*p* = 0.043 *	O	Partially Supported
		Depth	*p* = 0.986		
		Amount	*p* = 0.659		

Note: * *p* < 0.05, ** *p* < 0.01, *** *p* < 0.001

## Data Availability

The data presented in this study are openly available in Dropbox (https://www.dropbox.com/s/sv6yfoozmeandnj/Total%20data%20in%20paper_0817.xlsx?dl=0).

## Appendix A

**Table A1:** Measurement Items.

Variables	Measurement Items	References
**Disclosing Robot**	Home Companion-bots seemed to talk about what they were going through. Home Companion-bots seemed to be talking about themselves. Home Companion-bots seemed to tell what happened to them. Home Companion-bots seemed to talk about what they were experiencing	No ref.
**Involving User**	Home Companion-bots seemed to be talking about what I was going through. Home Companion-bots seemed to be talking about me. Home Companion-bots seemed to tell me what happened to me. Home Companion-bots seemed to tell me what I experienced.	No ref.
**Depth of User self-disclosure**	When I talked with Home Companion-bots, I intimately and fully revealed myself. When I talked with Home Companion-bots, I disclosed intimate, personal things about myself. When I talked with Home Companion-bots, I intimately disclosed who I really am.	Ma and Leung, 2006 [108]; Gibbs, Ellison, and Heino, 2006 [109]
**Amount of User self-disclosure**	When I talked to Home Companion-bots, I talked a lot about my feelings and thoughts. When I was talking to Home Companion-bots, I spoke for a long time about me. When I talked to Home Companion-bots, I did not talk much about myself. (reverse coding)	Ma and Leung, 2006 [108]; Gibbs, Ellison, and Heino, 2006 [109]
**Speech length**	This was measured by converting the voice response of the child to text and counting the number of characters in the text.	Moon (2000) [101]

## References

[1] Ng A. (2016). What Artificial Intelligence Can and Can’t Do Right Now. Harv. Bus. Rev..

[2] Nielsen (Smart) Speaking My Language: Despite Their Vast Capabilities, Smart Speakers Are All About the Music. https://www.nielsen.com/insights/2018/smart-speaking-my-language-despite-their-vast-capabilities-smart-speakers-all-about-the-music/.

[3] El Ayadi M., Kamel M.S., Karray F. (2011). Survey on speech emotion recognition: Features, classification schemes, and databases. Pattern Recognit..

[4] Ververidis D., Kotropoulos C. (2006). Emotional speech recognition: Resources, features, and methods. Speech Commun..

[5] Han K., Yu D., Tashev I. Speech emotion recognition using deep neural network and extreme learning machine. Proceedings of the Interspeech.

[6] Tian L., Moore J.D., Lai C. Emotion recognition in spontaneous and acted dialogues. Proceedings of the 2015 International Conference on Affective Computing and Intelligent Interaction (ACII).

[7] Fayek H.M., Lech M., Cavedon L. (2017). Evaluating deep learning architectures for speech emotion recognition. Neural Netw..

[8] Ministry of Gender Equality and Family R.o.K. (2015). National Survey on Families.

[9] Rajalakshmi J., Thanasekaran P. (2015). The effects and behaviours of home alone situation by latchkey children. Am. J. Nurs. Sci..

[10] Nomaguchi K.M., Milkie M.A., Bianchi S.M. (2005). Time strains and psychological well-being: Do dual-earner mothers and fathers differ?. J. Fam. Issues.

[11] Lee B., Cho M. (2011). The effects of after-school self-care on children’s development. J. Korean Soc. Child Welf..

[12] Ignatius E., Kokkonen M. (2007). Factors contributing to verbal self-disclosure. Nord. Psychol..

[13] Knox S., Hess S.A., Petersen D.A., Hill C.E. A qualitative analysis of client perceptions of the effects of helpful therapist self-disclosure in long-term therapy. Proceedings of the Annual Meeting of the Society for Psychotherapy.

[14] Barrett M.S., Berman J.S. (2001). Is psychotherapy more effective when therapists disclose information about themselves?. J. Consult. Clin. Psychol..

[15] Hill C.E., Knox S. (2001). Self-disclosure. Psychother. Theory Res. Pract. Train..

[16] Henretty J.R., Levitt H.M. (2010). The role of therapist self-disclosure in psychotherapy: A qualitative review. Clin. Psychol. Rev..

[17] Jourard S.M., Lasakow P. (1958). Some factors in self-disclosure. J. Abnorm. Soc. Psychol..

[18] Cozby P.C. (1973). Self-disclosure: A literature review. Psychol. Bull..

[19] Zhang R. (2017). The stress-buffering effect of self-disclosure on Facebook: An examination of stressful life events, social support, and mental health among college students. Comput. Hum. Behav..

[20] Miller K. (2003). Encyclopedia and Dictionary of Medicine, Nursing, and Allied Health.

[21] Watkins C.E. (1990). The effects of counselor self-disclosure: A research review. Couns. Psychol..

[22] Ziv-Beiman S. (2013). Therapist self-disclosure as an integrative intervention. J. Psychother. Integr..

[23] Henretty J.R., Currier J.M., Berman J.S., Levitt H.M. (2014). The impact of counselor self-disclosure on clients: A meta-analytic review of experimental and quasi-experimental research. J. Couns. Psychol..

[24] Levitt H.M., Minami T., Greenspan S.B., Puckett J.A., Henretty J.R., Reich C.M., Berman J.S. (2016). How therapist self-disclosure relates to alliance and outcomes: A naturalistic study. Couns. Psychol. Q..

[25] Collins N.L., Miller L.C. (1994). Self-disclosure and liking: A meta-analytic review. Psychol. Bull..

[26] Joinson A.N. (2001). Self-disclosure in computer-mediated communication: The role of self-awareness and visual anonymity. Eur. J. Soc. Psychol..

[27] Greene K., Derlega V.J., Mathews A. (2006). Self-disclosure in personal relationships. Camb. Handb. Pers. Relatsh..

[28] Bazarova N.N., Choi Y.H. (2014). Self-disclosure in social media: Extending the functional approach to disclosure motivations and characteristics on social network sites. J. Commun..

[29] Ruppel E.K. (2015). Use of communication technologies in romantic relationships: Self-disclosure and the role of relationship development. J. Soc. Pers. Relatsh..

[30] Kashian N., Jang J.-w., Shin S.Y., Dai Y., Walther J.B. (2017). Self-disclosure and liking in computer-mediated communication. Comput. Hum. Behav..

[31] Wheeless L.R. (1976). Self-disclosure and interpersonal solidarity: Measurement, validation, and relationships. Hum. Commun. Res..

[32] Forgas J.P. (2011). Affective influences on self-disclosure: Mood effects on the intimacy and reciprocity of disclosing personal information. J. Personal. Soc. Psychol..

[33] Gibson M.F. (2012). Opening up: Therapist self-disclosure in theory, research, and practice. Clin. Soc. Work J..

[34] Jourard S.M. (1971). Self-Disclosure. An experimental Analysis of the Transparent Self.

[35] Dindia K., Allen M. (1992). Sex differences in self-disclosure: A meta-analysis. Psychol. Bull..

[36] Mikulincer M., Nachshon O. (1991). Attachment styles and patterns of self-disclosure. J. Personal. Soc. Psychol..

[37] Turner R.N., Hewstone M., Voci A. (2007). Reducing explicit and implicit outgroup prejudice via direct and extended contact: The mediating role of self-disclosure and intergroup anxiety. J. Personal. Soc. Psychol..

[38] Lee K.-T., Noh M.-J., Koo D.-M. (2013). Lonely people are no longer lonely on social networking sites: The mediating role of self-disclosure and social support. Cyberpsychol. Behav. Soc. Netw..

[39] Powers A., Kiesler S., Fussell S., Torrey C. Comparing a computer agent with a humanoid robot. Proceedings of the ACM/IEEE International Conference on Human-Robot Interaction.

[40] Lucas G.M., Gratch J., King A., Morency L.-P. (2014). It’s only a computer: Virtual humans increase willingness to disclose. Comput. Hum. Behav..

[41] Pickard M.D., Roster C.A., Chen Y. (2016). Revealing sensitive information in personal interviews: Is self-disclosure easier with humans or avatars and under what conditions?. Comput. Hum. Behav..

[42] Kumazaki H., Warren Z., Swanson A., Yoshikawa Y., Matsumoto Y., Takahashi H., Sarkar N., Ishiguro H., Mimura M., Minabe Y. (2018). Can robotic systems promote self-disclosure in adolescents with autism spectrum disorder? A pilot study. Front. Psychiatry.

[43] Mumm J., Mutlu B. Human-robot proxemics: Physical and psychological distancing in human-robot interaction. Proceedings of the 6th International Conference on Human-Robot Interaction.

[44] Bethel C.L., Stevenson M.R., Scassellati B. Secret-sharing: Interactions between a child, robot, and adult. Proceedings of the 2011 IEEE International Conference on Systems, Man, and Cybernetics.

[45] Martelaro N., Nneji V.C., Ju W., Hinds P. Tell me more designing HRI to encourage more trust, disclosure, and companionship. Proceedings of the 2016 11th ACM/IEEE International Conference on Human-Robot Interaction (HRI).

[46] Hoffman G., Birnbaum G.E., Vanunu K., Sass O., Reis H.T. Robot responsiveness to human disclosure affects social impression and appeal. Proceedings of the 2014 ACM/IEEE International Conference on Human-Robot Interaction.

[47] Rosenthal-von der Pütten A.M., Krämer N.C., Herrmann J. (2018). The effects of humanlike and robot-specific affective nonverbal behavior on perception, emotion, and behavior. Int. J. Soc. Robot..

[48] Paine A.L., McCarthy Veach P., MacFarlane I.M., Thomas B., Ahrens M., LeRoy B.S. (2010). “What Would You Do if You Were Me?” Effects of Counselor Self-Disclosure Versus Non-disclosure in a Hypothetical Genetic Counseling Session. J. Genet. Couns..

[49] Hoffman-Graff M.A. (1977). Interviewer use of positive and negative self-disclosure and interviewer-subject sex pairing. J. Couns. Psychol..

[50] DeForest C., Stone G.L. (1980). Effects of sex and intimacy level on self-disclosure. J. Couns. Psychol..

[51] McCarthy P.R., Betz N.E. (1978). Differential effects of self-disclosing versus self-involving counselor statements. J. Couns. Psychol..

[52] Danish S.J., D’Augelli A.R., Brock G.W. (1976). An evaluation of helping skills training: Effects on helpers’ verbal responses. J. Couns. Psychol..

[53] Altman I., Taylor D.A. (1973). Social Penetration: The Development of Interpersonal Relationships.

[54] Laurenceau J.-P., Barrett L.F., Rovine M.J. (2005). The interpersonal process model of intimacy in marriage: A daily-diary and multilevel modeling approach. J. Fam. Psychol..

[55] Sprecher S.K., Hendrick S.S. (2004). Self-disclosure in intimate relationships: Associations with individual and relationship characteristics over time. J. Soc. Clin. Psychol..

[56] Breazeal C. (2003). Emotion and sociable humanoid robots. Int. J. Hum. Comput. Stud..

[57] Kumar N., Benbasat I. (2002). Para-social presence and communication capabilities of a web site: A theoretical perspective. e-Service.

[58] Kang S.H., Gratch J. (2010). Virtual humans elicit socially anxious interactants’ verbal self-disclosure. Comput. Animat. Virtual Worlds.

[59] Huang L., Morency L.-P., Gratch J. Virtual Rapport 2. 0. In Proceedings of the International workshop on Intelligent Virtual Agents.

[60] Kang S.-H., Gratch J., Sidner C., Artstein R., Huang L., Morency L.-P. Towards building a virtual counselor: Modeling nonverbal behavior during intimate self-disclosure. Proceedings of the 11th International Conference on Autonomous Agents and Multiagent Systems-Volume 1.

[61] Zhao R., Papangelis A., Cassell J. Towards a dyadic computational model of rapport management for human-virtual agent interaction. Proceedings of the International Conference on Intelligent Virtual Agents.

[62] Aggarwal P., McGill A.L. (2007). Is that car smiling at me? Schema congruity as a basis for evaluating anthropomorphized products. J. Consum. Res..

[63] Epley N., Waytz A., Cacioppo J.T. (2007). On seeing human: A three-factor theory of anthropomorphism. Psychol. Rev..

[64] Nass C.I., Moon Y., Morkes J. (1997). Computers are social actors: A review of current. Hum. Values Des. Comput. Technol..

[65] Moon Y., Nass C. (1996). How “real” are computer personalities? Psychological responses to personality types in human-computer interaction. Commun. Res..

[66] Nass C., Moon Y., Fogg B.J., Reeves B., Dryer D.C. (1995). Can computer personalities be human personalities?. Int. J. Hum. Comput. Stud..

[67] McCarthy Veach P. (2011). Reflections on the meaning of clinician self-reference: Are we speaking the same language?. Psychotherapy.

[68] Reis H.T. (2018). Intimacy as an interpersonal process. Relationships, Well-Being and Behaviour.

[69] Forest A.L., Wood J.V. (2011). When partner caring leads to sharing: Partner responsiveness increases expressivity, but only for individuals with low self-esteem. J. Exp. Soc. Psychol..

[70] Sultan S., Chaudry H. (2008). Gender-based differences in the patterns of emotional self-disclosure. Pak. J. Psychol. Res..

[71] Richard E., Emerson R. (1976). Social exchange theory. Annu. Rev. Sociol..

[72] Rotenberg K.J., Chase N. (1992). Development of the reciprocity of self-disclosure. J. Genet. Psychol..

[73] Solano C.H., Dunnam M. (1985). Two’s company: Self-disclosure and reciprocity in triads versus dyads. Soc. Psychol. Q..

[74] Nass C., Moon Y. (2000). Machines and mindlessness: Social responses to computers. J. Soc. Issues.

[75] Lee K.M., Nass C. (2004). The multiple source effect and synthesized speech: Doubly-disembodied language as a conceptual framework. Hum. Commun. Res..

[76] Biocca F. (1997). The cyborg’s dilemma: Progressive embodiment in virtual environments. J. Comput.-Mediat. Commun..

[77] Sundar S.S. (2008). The MAIN Model: A Heuristic Approach to Understanding Technology Effects on Credibility.

[78] Kim K.J. (2016). Interacting socially with the Internet of Things (IoT): Effects of source attribution and specialization in human–IoT interaction. J. Comput. -Mediat. Commun..

[79] Such J.M., Espinosa A., García-Fornes A. (2014). A survey of privacy in multi-agent systems. Knowl. Eng. Rev..

[80] Chung H., Iorga M., Voas J., Lee S. (2017). Alexa, can I trust you?. Computer.

[81] Venturebeat. IoT Device Pairing Raises Privacy Concerns for Home AI. https://venturebeat.com/ai/iot-device-pairing-raises-privacy-concerns-for-home-ai/.

[82] Lee H., Wong S.F., Oh J., Chang Y. (2019). Information privacy concerns and demographic characteristics: Data from a Korean media panel survey. Gov. Inf. Q..

[83] Blank G., Bolsover G., Dubois E. A new privacy paradox: Young people and privacy on social network sites. Proceedings of the Prepared for the Annual Meeting of the American Sociological Association.

[84] Kezer M., Sevi B., Cemalcilar Z., Baruh L. (2016). Age differences in privacy attitudes, literacy and privacy management on Facebook. Cyberpsychol. J. Psychosoc. Res. Cyberspace.

[85] Park Y.J. (2013). Digital literacy and privacy behavior online. Commun. Res..

[86] Paine C., Reips U.-D., Stieger S., Joinson A., Buchanan T. (2007). Internet users’ perceptions of ‘privacy concerns’ and ‘privacy actions’. Int. J. Hum.-Comput. Stud..

[87] Youn S. (2005). Teenagers’ perceptions of online privacy and coping behaviors: A risk–benefit appraisal approach. J. Broadcast. Electron. Media.

[88] Solove D.J. (2005). A taxonomy of privacy. U. Pa. L. Rev..

[89] Malhotra N.K., Kim S.S., Agarwal J. (2004). Internet users’ information privacy concerns (IUIPC): The construct, the scale, and a causal model. Inf. Syst. Res..

[90] Eastlick M.A., Lotz S.L., Warrington P. (2006). Understanding online B-to-C relationships: An integrated model of privacy concerns, trust, and commitment. J. Bus. Res..

[91] Ware J.N., Dillman Taylor D. (2014). Concerns about confidentiality: The application of ethical decision-making within group play therapy. Int. J. Play Ther..

[92] Sprecher S., Treger S., Wondra J.D., Hilaire N., Wallpe K. (2013). Taking turns: Reciprocal self-disclosure promotes liking in initial interactions. J. Exp. Soc. Psychol..

[93] Lee S., Choi J. (2017). Enhancing user experience with conversational agent for movie recommendation: Effects of self-disclosure and reciprocity. Int. J. Hum.-Comput. Stud..

[94] Krasnova H., Spiekermann S., Koroleva K., Hildebrand T. (2010). Online social networks: Why we disclose. J. Inf. Technol..

[95] Gouldner A.W. (1960). The norm of reciprocity: A preliminary statement. Am. Sociol. Rev..

[96] Faul F., Erdfelder E., Lang A.-G., Buchner A. (2007). G* Power 3: A flexible statistical power analysis program for the social, behavioral, and biomedical sciences. Behav. Res. Methods.

[97] Lee J.D., Brown T.L., Caven B., Haake S., Schmidt K. Does a speech-based interface for an in-vehicle computer distract drivers? In Proceedings of the World Congress on Intelligent Transport System, San Francisco, CA, USA, 6–9 November 2000.

[98] Jung S., Sandor C., Wisniewski P.J., Hughes C.E. Realme: The influence of body and hand representations on body ownership and presence. Proceedings of the 5th Symposium on Spatial User Interaction.

[99] Caine K. Local standards for sample size at CHI. Proceedings of the 2016 CHI Conference on Human Factors in Computing Systems.

[100] Cowie R., Douglas-Cowie E., Tsapatsoulis N., Votsis G., Kollias S., Fellenz W., Taylor J.G. (2001). Emotion recognition in human-computer interaction. IEEE Signal Process. Mag..

[101] Moon Y. (2000). Intimate exchanges: Using computers to elicit self-disclosure from consumers. J. Consum. Res..

[102] Wheeless L.R., Grotz J. (1976). Conceptualization and measurement of reported self-disclosure. Hum. Commun. Res..

[103] Wheeless L.R. (1978). A follow-up study of the relationships among trust, disclosure, and interpersonal solidarity. Hum. Commun. Res..

[104] Posey C., Lowry P.B., Roberts T.L., Ellis T.S. (2010). Proposing the online community self-disclosure model: The case of working professionals in France and the UK who use online communities. Eur. J. Inf. Syst..

[105] Ko H.-C. (2013). The determinants of continuous use of social networking sites: An empirical study on Taiwanese journal-type bloggers’ continuous self-disclosure behavior. Electron. Commer. Res. Appl..

[106] Liu Z., Min Q., Zhai Q., Smyth R. (2016). Self-disclosure in Chinese micro-blogging: A social exchange theory perspective. Inf. Manag..

[107] Hollenbaugh E.E., Ferris A.L. (2015). Predictors of honesty, intent, and valence of Facebook self-disclosure. Comput. Hum. Behav..

[108] Ma M.L.-Y., Leung L. (2006). Unwillingness-to-communicate, perceptions of the Internet and self-disclosure in ICQ. Telemat. Inform..

[109] Gibbs J.L., Ellison N.B., Heino R.D. (2006). Self-presentation in online personals: The role of anticipated future interaction, self-disclosure, and perceived success in Internet dating. Commun. Res..

[110] Bagozzi R.P., Yi Y. (1988). On the evaluation of structural equation models. J. Acad. Mark. Sci..

[111] Chin W.W. (1998). The partial least squares approach to structural equation modeling. Mod. Methods Bus. Res..

[112] Fornell C., Larcker D.F. (1981). Evaluating structural equation models with unobservable variables and measurement error. J. Mark. Res..

[113] Gefen D., Straub D., Boudreau M.-C. (2000). Structural equation modeling and regression: Guidelines for research practice. Commun. Assoc. Inf. Syst..

[114] Segars A.H., Grover V. (1998). Strategic information systems planning success: An investigation of the construct and its measurement. MIS Q..

[115] Bakeman R. (2005). Recommended effect size statistics for repeated measures designs. Behav. Res. Methods.

[116] Barnett J.E. (2011). Psychotherapist self-disclosure: Ethical and clinical considerations. Psychotherapy.

[117] Peterson Z.D. (2002). More than a mirror: The ethics of therapist self-disclosure. Psychother. Theory Res. Pract. Train..

[118] Sturges J.W. (2012). Use of therapist self-disclosure and self-involving statements. Behav. Ther..

[119] Audet C.T., Everall R.D. (2010). Therapist self-disclosure and the therapeutic relationship: A phenomenological study from the client perspective. Br. J. Guid. Couns..

